# Supplementary material for: Predictive factors of distant metastasis in surgically treated HPV-positive tonsil cancer
Source: PLoS One. 2023 Mar 21;18(3):e0283368. doi: 10.1371/journal.pone.0283368 (PMC10030005; doi:10.1371/journal.pone.0283368)
Supplement: Attachment — Submitted filename: Responses to Reviewers.docx [file pone.0283368.s001.docx]

**Reviewer #1:** “Predictive factors of distant metastasis in surgically treated HPV positive tonsil cancer” finds that in a retrospective review of HPV positive oropharyngeal SCC undergoing primary surgery, positive margins and advanced T stage are independent predictors of delayed distant metastases. In the same cohort, distant metastasis and margin involvement were predictive of worse survival outcomes.

This study is one of the largest reviews to date specifically looking at delayed distant metastasis in HPV mediated oropharyngeal SCC undergoing primary surgery. Factors including TNM classification, adjuvant therapy, margin involvement, margin type, and histopathologic characteristics were analyzed.

There remain some points that might be clarified:

**Major:**
**1) The means by which patients were found to have distant metastasis remains unclear. Did all patients undergo repeat PET at certain intervals after treatment? Additional clarity on surveillance protocols would be helpful**

**Response:**

Thank you for your suggestion.

In our center, chest CT, abdomen-pelvis CT, bone scan, and primary site MRI were performed every 6 months after the end of treatment for 5 years. If new symptoms such as pain are found in the period of follow-up, an image study is performed on the relevant area to check for abnormalities. If a lesion suspected of distant metastasis is found, additional tests such as biopsy or PET-CT are performed to confirm distant metastasis. Patients with images demonstrating evidence of distant metastatic disease were re-staged and pathologic confirmation for site of distant metastasis had tried in available cases.

We have included this information in the Method section.

**2) For the 5 year disease specific survival, is the calculation for distant metastasis from time of treatment or from time of diagnosis of distant metastasis?**

**Response:**

Thank you for comment.

Other study sets the end point differently. McBride set the time to distant metastasis as the time from completion of surgery to the first radiographic evidence of distant metastatic disease. [McBride] In the Ann’s study, the primary end point was overall survival, defined as the time from randomization to death. Secondary end points included progression-free survival, defined as the time from randomization to death or the first documented relapse, which was categorized as local–regional disease (tumor at the primary site or regional nodes) or distant metastases.

This study calculated the follow-up period from the day of surgery. Because, the subject of this study is that patients who have not received neoadjuvant and observed after surgery are subjects.

*Ref)*

S.M. McBride et al. / Long-term survival after distant metastasis in patients with oropharyngeal cancer / Oral Oncology 50 (2014) 208–212

Ang KK, Harris J, Wheeler R, Weber R, Rosenthal DI, Nguyen-Tân PF, et al. (2010) Human papillomavirus and survival of patients with oropharyngeal cancer. N Engl J Med. 363:24-35

We have included this information in the Method section.

**3) A table of characteristics in each patient with distant metastasis**

**Response:**

Thank you for suggestion.

Of 12 (15.7%) patients who experienced a distant failure, two patients had both locoregional and distant failure with all lung metastasis. The median time to distant metastases was 32 months (range, 7–96 months). Two patients showed early distant metastasis (within 1 year). Sites of distant metastasis included lung (n = 10, 83.3%), liver (n = 1, 8.3%), and brain (n = 1, 8.3%). Ten (83.3%) patients had undergone treatment for their distant metastasis, including six patients who received chemotherapy alone, two patients who received chemoradiation, and two patients who received both surgery and chemotherapy. Surgical intervention included wedge resections for pulmonary metastases in two patients. 4 patients (33.3%) had smoking history, as same, 4 patients had alcohol history.

For margin status, 6 patients (50%) showed close margin (1mm< <5mm) and 6 patients showed negative margin (5mm <). An infiltrative margin type was observed in 9 patients (75%) and 3 patients (25%) showed expanding margin type. 7 patients (58.3%) had lympho-vascular invasion and 1 patient (8.3%) had perineural invasion. The mean depth of invasion was 15.16 mm, and ENE was observed in 4 patients (25%).

We have included this information in the result section and supplementary table (Table 2.)

Table 2**. Characteristics of patients who developed distant metastasis**

| Patient no. | Site of recurrence | T | N | Adjuvant modality | Salvage modality | Time to distant metastasis (month) | Survival | Smoking | Alcohol |
| --- | --- | --- | --- | --- | --- | --- | --- | --- | --- |
| 1 | Lung | 2 | 1 | CCRT | Chemo | 18 | No | No | No |
| 2 | Lung | 3 | 0 | None | Surgery, Chemo | 38 | No | No | No |
| 3 | Lung | 3 | 1 | CCRT | Chemo | 35 | No | Yes | Yes |
| 4 | Liver | 3 | 2 | CCRT | Chemo | 11 | YES | Yes | Yes |
| 5 | Lung | 1 | 1 | RT | Refuse | 96 | No | Yes | Yes |
| 6 | Lung | 2 | 2 | CCRT | Chemo, Radiation | 46 | No | No | No |
| 7 | Lung | 2 | 1 | RT | Surgery, Chemo | 61 | No | No | No |
| 8 | Brain | 3 | 1 | RT | Refuse | 58 | No | Yes | Yes |
| 9 | Lung | 3 | 2 | CCRT | Chemo, Radiation | 30 | No | No | No |
| 10 | Lung | 3 | 2 | CCRT | Chemo | 19 | No | No | No |
| 11 | Lung | 2 | 1 | RT | Chemo | 22 | YES | No | No |
| 12 | Lung | 3 | 1 | RT | Chemo | 13 | YES | No | No |

**Characteristics of patients who developed distant metastasis (continued)**

| Patient no. | Surgical margin status | Lymphatic invasion | Vascular invasion | Perineural invasion | DOI (mm) | ENE | Margin type |
| --- | --- | --- | --- | --- | --- | --- | --- |
| 1 | Close | No | No | No | 20 | No | Infiltrative |
| 2 | Close | No | No | No | 17 | Yes | Infiltrative |
| 3 | Negative | Yes | No | No | 25 | Yes | Infiltrative |
| 4 | Close | Yes | No | No | 21 | No | Expanding |
| 5 | Negative | Yes | No | No | 16 | No | Expanding |
| 6 | Negative | No | No | No | 11 | No | Both |
| 7 | Negative | Yes | Yes | No | 8 | No | Both |
| 8 | Negative | No | No | No | 20 | No | Infiltrative |
| 9 | Close | Yes | No | No | 14 | Yes | Infiltrative |
| 10 | Close | Yes | No | Yes | 16 | No | Expanding |
| 11 | Close | No | No | No | 13 | No | Infiltrative |
| 12 | Negative | Yes | Yes | No | 1 | Yes | Both |

DOI: Depth of invasion, ENE: Extra nodal extension

CCRT: Concurrent-chemoradiation therapy, RT: Radiation therapy

**Minor:**

**1) In the background, there is a statement, “This could be explained by differences in men's smoking, sexual behavior, and sexual organ structure.” Please clarify and cite**

**Response:**

Thank you for your comment.

The incidence of head and neck cancers overall has declined in recent years, consistent with the decrease in tobacco use. By contrast, incidence of HPV-associated oropharyngeal cancer seems to be rising. In particular, the prevalence is increasing rapidly in young men.[Shiboski] This effect may be attributable to changes in sexual norms (more oral sex partners or oral sex at an earlier age in recent than past generations) combined with fewer tobacco-related cancers in young cohorts, making the outcomes of HPV-positive cancers more apparent.[Marur] In addition, HPV prevalence in cervix rather than penile tissue might boost the chances of HPV infection when performing oral sex on a woman, contributing to the higher rate of HPV-associated oropharyngeal cancer in men.[Marur] In view of the importance of tobacco use in HPV related OPC, there are some study that reported oral HPV infection is common in smokers and non-smokers and it is an important cause of oropharyngeal cancer in both groups.[Smith ; Gillison] Although a higher proportion of individuals with HPV-positive compared with HPV-negative tumors are non-smokers or neither smoke nor drink alcohol, many with HPV-positive disease have a history of alcohol and tobacco use. This finding underscores that HPV-associated malignant disease not only arises in people who do not smoke or drink alcohol but also occurs in people with the traditional risk factors of tobacco and alcohol use.

We have included this information with citation in the Background section.

*Ref)*

Shiboski CH, Schmidt BL, Jordan RC. Tongue and tonsil carcinoma: increasing trends in the U.S. population ages 20–44 years. Cancer 2005; 103: 1843–49

Marur S, D'Souza G, Westra WH, Forastiere AA. (2010) HPV-associated head and neck cancer: a virus-related cancer epidemic. Lancet Oncol. 11:781-789

Smith EM, Ritchie JM, Summersgill KF, et al. Age, sexual behavior and human papillomavirus infection in oral cavity and oropharyngeal cancers. Int J Cancer 2004; 108: 766–72

Gillison ML, D’Souza G, Westra W, et al. Distinct risk factor profi les for human papillomavirus type 16-positive and human papillomavirus type 16-negative head and neck cancers. J Natl Cancer Inst 2008; 100: 407–20

**2) “Since 1970, HPV associated head and neck cancer is increasing more rapidly in men. The average age of diagnosis is 55 to 60 years, which is about 10 years younger than HPV-associated head and neck cancer”. Clarify HPV and non-HPV cohorts**

**Response:**

Thank you for suggestion,

The sentence corrected to “Since 1970, HPV associated head and neck cancer is increasing more rapidly in men. The average age of diagnosis is 55 to 60 years, which is about 10 years younger than HPV-unrelated head and neck cancer”

We have corrected this sentence in the manuscript.

**3) For patients with distant metastasis, please consider including treatments received and tumor characteristics as a separate cohort in “Patients’ characteristics and surgical parameters” section**

**Response:**

Thank you for your thoughtful comment.

Following your suggestion, a brief characteristic of cohort with distant metastasis included in “Risk factors of distant metastasis” section was further reinforced and moved to the “patient’s characteristics and surgical parameters” section as follows,

Of 12 (15.7%) patients who experienced a distant failure, two patients had both locoregional and distant failure with all lung metastasis. The median time to distant metastases was 32 months (range, 7–96 months). Two patients showed early distant metastasis (within 1 year). Sites of distant metastasis included lung (n = 10, 83.3%), liver (n = 1, 8.3%), and brain (n = 1, 8.3%). Ten (83.3%) patients had undergone treatment for their distant metastasis, including six patients who received chemotherapy alone, two patients who received chemoradiation, and two patients who received both surgery and chemotherapy. Surgical intervention included wedge resections for pulmonary metastases in two patients. 4 patients (33.3%) had smoking history, as same, 4 patients had alcohol history.

For margin status, 6 patients (50%) showed close margin (1mm< <5mm) and 6 patients showed negative margin (5mm <). An infiltrative margin type was observed in 9 patients (75%) and 3 patients (25%) showed expanding margin type. 7 patients (58.3%) had lympho-vascular invasion and 1 patient (8.3%) had perineural invasion. The mean depth of invasion was 15.16 mm, and ENE was observed in 4 patients (25%).

We have included this in [Patients’ characteristics and surgical parameters] section.

**4) Please cite any past papers looking at margin type and their conclusions**

**Response:**

Thank you for suggestion.

Authors could not find studies that analyzed the margin type (infiltrative vs. expanding) in HPV related/non related OPC cancer. However, studies on the margin type of malignancy in other sites such as thyroid, colorectal could be found. Kim et al. reported clinical outcomes of papillary thyroid carcinoma according to tumor margin type. Kim found infiltrative type margin was associated with significantly higher rates of tumor multiplicity, extrathyroidal extension, coexistence of lymphocytic thyroiditis, and metastasis to lateral neck lymph nodes than the expanding type margin.[Kim] Garcia-Solano et al. published a paper on pathologic features such as tumor budding and invasive margin type in colorectal adenocarcinoma. He found that the infiltrative margin type was a worse prognostic factor for 5-year survival than the expanding type in serrated adenocarcinoma.[Garcia-Solano]

Based on these studies, the infiltrative margin type may act as a predictor of poor oncologic outcome, and it is consistent with result of this study.

We have included this in discussion section

*Ref)*

Kim et, al. Tumor Margin Histology Predicts Tumor Aggressiveness in Papillary Thyroid Carcinoma: A Study of 514 Consecutive Patients. J Korean Med Sci 2011; 26: 346-351

Garcı´a-Solano et, al. Tumour budding and other prognostic pathological features at invasive margins in serrated colorectal adenocarcinoma: a comparative study with conventional carcinoma, Histopathology, 2011, 59: 1046–1056

**5) Were there any patients in the study that did not receive the NCCN standard of care treatment recommendations?**

**Response:**

The treatment protocol at this center is determined on the tumor board based on the NCCN guide line. The cohort included in this study included only the cases where observation or adjuvant therapy was performed after surgical treatment among the NCCN standard protocols. All 76 patients were treated according to the NCCN guideline.

We have included this information in the Method section.

**6) “It can be inferred that the invasiveness of infiltrative tumor margin causing distant metastasis is stronger.” This is only significant on univariate analysis so consider not including this statement**

**Response:**

Thank you for suggestion.

Following your comment, the sentence was removed from the original manuscript.

**7) “In conclusion, the current study is the largest and the most robust analysis up to date to identify”. Delete “up”**

**Response:**

Thank you for suggestion.

Following your comment, the sentence was corrected.

**Reviewer #2**: This is a retrospective review examining distant failures following primary surgical management of 76 patients with HPV-mediated tonsil cancer treated at a single institution over a 12 year period. The study aims to identify risk factors for distant failures after surgical management, which the authors note is currently lacking in the literature for patients not having undergone neoadjuvant treatment. All patients underwent primary surgical management with risk-adjusted adjuvant treatment. None had been previously treated prior to surgery. Overall survival and disease specific survival for all-comers in this cohort was excellent. Advanced T classification and positive marginal status were shown to be independent predictors of distant metastasis in this cohort by multivariate analysis. Infiltrative margin type was also associated with distant metastasis by univariate analysis only. Moreover, distant metastasis and positive marginal status were associated with significantly worsened 5-year disease-specific survival. The authors conclude that this is the largest cohort of its type, providing crucial risk stratification data and information for patient counseling in this population.


**Major Points**

**1. The authors note that other studies have suggested that the tonsillar subsite has high rates of distant failure, but they might clarify why the tonsillar subsite was exclusively examined rather than examining both tonsillar and base of tongue subsites (or alternatively all oropharyngeal subsites). This may strengthen the study by increasing the size of the cohort and also provide additional data about the other subsites.**

**Response:**

Thank you for your thoughtful comment.

Most of the other studies have reported recurrence or survival of the oropharynx unit, not each sub-site. Wendt reported recurrence and survival according to the sub-site of HPV-positive OPC. In his study, the primary sites consisted of tonsil (63%), base of tongue (22%), and other oropharynx (15%). In his study, in the case of tonsil, 35% of patients experienced distant failure, which is a result of examining the distant failure rate regardless of treatment modality.[Wendt] The treatment protocol at this center is determined on the tumor board based on the NCCN guide line. The cohort included in this study included only the cases where observation or adjuvant therapy was performed after surgical treatment among the NCCN standard protocols. In the case of base of tongue lesions, our center recommends neoadjuvant chemotherapy or CCRT rather than initial surgery with intend to improve the patient's swallowing functional outcome. Therefore, patients with base of tongue lesions were not included in this study.

We have included this information in the Method section.

*Ref.)*

Wendt M, Hammarstedt-Nordenvall L, Zupancic M, Friesland S, Landin D, Munck-Wikland E, et al. (2021) Long-Term Survival and Recurrence in Oropharyngeal Squamous Cell Carcinoma in Relation to Subsites, HPV, and p16-Status. Cancers (Basel) 2021; 13:2553.

**2. Nearly half of the patients who underwent surgery in this cohort required both adjuvant chemotherapy and radiation. It may be useful to discuss surgical selection criteria at the authors’ institution. Triple modality therapy may be unavoidable in a subset of patients with unanticipated, unresectable positive margins or unexpected extracapsular extension. However, many providers/tumor boards are likely to advocate forgoing surgery in patients who are at high risk to require triple modality therapy if subjected to surgery. While the authors report excellent overall survival comparable with other HPV-mediated cohorts, there may have been some missed opportunities to limit treatment-related morbidities in the patients undergoing triple modality treatment. How do the rates of positive margins and proportion of patients undergoing triple modality treatment in this cohort compare to those reported in other studies?**

**Response:**

Thank you for comment.

The treatment protocol at this center is determined on the tumor board based on the NCCN guide line. In the case of advanced stage, the treatment modality is determined on the tumor board in consideration of the functional outcome after surgery, and after sufficient explanation to the patient, the treatment method is determined by respecting the patient's choice. The cohort included in this study included only the cases where observation or adjuvant therapy was performed after surgical treatment among the NCCN standard protocols. All 76 patients were agreed to initial surgical treatment.

Regarding surgical margin, positive margin in 16 (21%), negative margin in 56 (73.6%), and close margin in 4 (5.2%) patients were found. Among patients with positive margin, 13 out of 16 (81.2%) got concurrent chemo-radiation therapy (CCRT), 2 patients (1.6%) got radiation alone therapy, and 1 patient was observed with active surveillance (patient’s refusal to adjuvant therapy). Choi et al. published a study on the prognosis according to lymph node ratio after upfront surgery in HPV-positive OPC patients, and presented positive margin and ENE as main indications for adjuvant therapy. In her study, a positive margin was found in 25 patients (27.7%) out of 90 patients, and adjuvant RT was performed in 26 patients (28.9%) and CCRT was performed in 47 patients (52.2%). Roden et al. published a study comparing outcomes of upfront surgery with adjuvant therapy or CCRT in advanced stage HPV-positive tonsil cancer patients using a national data base. Surgery was performed in 8768 patients, and positive margins were observed in 2762 (31.5%). Among cohort with positive margin, adjuvant RT was performed in 25.9% and CCRT was performed in 35.2%. These are summarized in the table below.

| Ratio | The present study | Choi et, al. | Roden et, al. |
| --- | --- | --- | --- |
| Positive margin | 21% | 27.7% | 31.5% |
| Adjuvant RT | 1.6% | 28.9% | 25.9% |
| Adjuvant CCRT | 81.2% | 52.2% | 35.2% |

In this study, the proportion of patients who showed a positive margin was smaller than in the other two studies, and the proportion of patients who underwent CCRT was higher than in the other two studies. This is thought to be influenced by the preference of this center tumor board.

We have included this information in the Method & Result section.

*Ref.)*

Choi et, al. Importance of lymph node ratio in HPV-related oropharyngeal cancer patients treated with surgery and adjuvant treatment, PLOS ONE, 2021

Roden et, al. Triple-Modality Treatment in Patients With Advanced Stage Tonsil Cancer, 2017, cancer

**3. The effect of marginal type is reported with respect to distant metastasis. Did the authors examine the relationship of infiltrative vs expanding marginal types on survival as well?**

**Response:**

Thank you for comment.

Multivariate survival analysis considering the margin type is presented in Table 3 and it was not statistically significant (OR: 0.58, 95% CI: 0.08-4.00, *p* = 0.55). Following your comment, a disease specific survival curve was created for each margin type. 5-yr DSS rate of the infiltrative type was 60.5%, and the expanding type showed 83.6%. (p=0.14)

We have included this information in the result & additional figure

Additional figure (Figure 2. Kaplan–Meier figures of disease-specific survival in patients with HPV positive tonsil cancer according to margin type)

**4. Were any patients treated with immunotherapy after the diagnosis of their distant metastasis?**

**Response:**

Distant metastasis occurred in 12 patients in this study. Two patients did not want active treatment after diagnosis of distant failure, and unfortunately, the remaining 10 patients were unable to perform immunotherapy due to economic problems (insurance coverage, etc.).

**Minor Points:**
**1. (Page 9, paragraph 1, lines 2-3) P16 is a surrogate marker for HPV-mediated carcinoma rather than a “type.” The authors should clarify this.**

**Response:**

Thank you for comment.

In present study, HPV status of each patient was confirmed by in situ hybridization for HPV DNA or by strong and diffuse staining for p16. There was an error in the choice of terminology. “P16” in the manuscript refers to high risk HPV 16 type. Following your comment, that sentence was corrected from “Among HPV types, p16 type is the main cause” to “Among HPV types, HPV 16 type is the main cause and it is characterized by high levels of p16 marker” (Citation is the same because it is referenced in the same paper)

We have included this information in the Background section.

**2. (Page 9, paragraph 1, lines 5-6) the authors talk about HPV-associated cancer in men in the previous sentence and then reference diagnosis being 10 years earlier than HPV-associated cancer. Please clarify, since it seems like an error (are the authors trying to compare this to the age of diagnosis in women, for patients with HPV-negative carcinoma, or something else?).**

**Response:**

Thank you for comment.

The meaning of the sentence was insufficiently made, and there was a mistake in the choice of terminology.

The sentence corrected to “Since 1970, HPV associated head and neck cancer is increasing more rapidly in men. The average age of diagnosis is 55 to 60 years, which is about 10 years younger than HPV-unrelated head and neck cancer”

Additionally, the incidence of head and neck cancers overall has declined in recent years, consistent with the decrease in tobacco use. By contrast, incidence of HPV-associated oropharyngeal cancer seems to be rising. In particular, the prevalence is increasing rapidly in young men.[Shiboski] This effect may be attributable to changes in sexual norms (more oral sex partners or oral sex at an earlier age in recent than past generations) combined with fewer tobacco-related cancers in young cohorts, making the outcomes of HPV-positive cancers more apparent.[Marur] In addition, HPV prevalence in cervix rather than penile tissue might boost the chances of HPV infection when performing oral sex on a woman, contributing to the higher rate of HPV-associated oropharyngeal cancer in men.[Marur] In view of the importance of tobacco use in HPV related OPC, there are some study that reported oral HPV infection is common in smokers and non-smokers and it is an important cause of oropharyngeal cancer in both groups.[Smith ; Gillison] Although a higher proportion of individuals with HPV-positive compared with HPV-negative tumors are non-smokers or neither smoke nor drink alcohol, many with HPV-positive disease have a history of alcohol and tobacco use. This finding underscores that HPV-associated malignant disease not only arises in people who do not smoke or drink alcohol but also occurs in people with the traditional risk factors of tobacco and alcohol use.

*Ref)*

Shiboski CH, Schmidt BL, Jordan RC. Tongue and tonsil carcinoma: increasing trends in the U.S. population ages 20–44 years. Cancer 2005; 103: 1843–49

Marur S, D'Souza G, Westra WH, Forastiere AA. (2010) HPV-associated head and neck cancer: a virus-related cancer epidemic. Lancet Oncol. 11:781-789

Smith EM, Ritchie JM, Summersgill KF, et al. Age, sexual behavior and human papillomavirus infection in oral cavity and oropharyngeal cancers. Int J Cancer 2004; 108: 766–72

Gillison ML, D’Souza G, Westra W, et al. Distinct risk factor profi les for human papillomavirus type 16-positive and human papillomavirus type 16-negative head and neck cancers. J Natl Cancer Inst 2008; 100: 407–20

We have included this information in the Background section.

**3. (Page 9, paragraph 1, lines 15-16) Please correct “16 negative”**

**Response:**

Thank you for comment.

Following your comment, “16 negative” was corrected to “p16 negative”

We have corrected this information in the Background section.

**4. (Page 13, paragraph 1, lines 3-4) The authors reference “advanced n-stage” as including N2 or N3. However, the AJCC 8 nodal classification system does not have a pathological N3 designation. Does this mean that the N-classification of these tumors was determined clinical evaluation alone or by combining available clinical and pathological data?**

**Response:**

Thank you for comment.

There was a mistake in the choice of terminology. As you commented, nodal stage system of HPV-positive oropharyngeal cancer is consisted of N0, N1, and N2. We deleted “N3” from manuscript. Table 1. contains information of patient’s N classification and we confirmed it was configured correctly with the nodal staging system.

We have corrected this information in the result section.

**5. (Page 14, paragraph 2) when reporting the univariate analysis results, it is not always clear which variables correspond to which of the percentages in parentheses. For example, which margin type had a significantly higher association with distant metastasis?**

**Response:**

Thank you for comment.

Following your comment, variables corresponding to each rate value are indicated in the manuscript. (Advanced T stage: 53.3%, positive margin: 37.5%, infiltrative margin type: 15%)

We have included this information in the Result section.

**6. The overall writing style, punctuation, and grammar should be closely examined. The authors are not consistent with indentation between various paragraphs and sections. There is also a variety of minor grammatical errors.**

**Response:**

Thank you for suggestion.

The authors commissioned the native speaker again for the overall English proofreading.
