# Supplementary material for: Predictive factors of distant metastasis in surgically treated HPV-positive tonsil cancer
Source: PLoS One. 2023 Mar 21;18(3):e0283368. doi: 10.1371/journal.pone.0283368 (PMC10030005; doi:10.1371/journal.pone.0283368)
Supplement: Attachment — Submitted filename: Response to Reviewers - revision 2.docx [file pone.0283368.s002.docx]

**Editor**

**1) Please review your reference list to ensure that it is complete and correct. If you have cited papers that have been retracted, please include the rationale for doing so in the manuscript text, or remove these references and replace them with relevant current references. Any changes to the reference list should be mentioned in the rebuttal letter that accompanies your revised manuscript. If you need to cite a retracted article, indicate the article’s retracted status in the References list and also include a citation and full reference for the retraction notice.**

**Response:**

Thank you for your suggestion. As the editor requested, we reviewed the current status of the papers in the reference list and the reference list has been revised as follows.

| Previous Citation No. | Citation | Change |
| --- | --- | --- |
| 4 | Marur S, D'Souza G, Westra WH, Forastiere AA. HPV-associated head and neck cancer: a virus-related cancer epidemic. The lancet oncology. 2010; 11:781-789. | Deleted due to duplication with citation number 2. |
| 17 | Haberal I, Celik H, Göçmen H, Akmansu H, Yörük M, Ozeri C. Which is important in the evaluation of metastatic lymph nodes in head and neck cancer: palpation, ultrasonography, or computed tomography? Otolaryngol Head Neck Surg. 2004; 130:197-201. doi: 10.1016/j.otohns.2003.08.025 PMID: 14990916 | Due to the improper form of the citation, endnote file was changed to a new one. |

**2) The authors have improved their manuscript and answered the reviewer comments. There are numerous grammatical errors (improper tense, etc.) throughout the manuscript that would need to be edited, preferably by a professional editor, before acceptance.**

**Response:**

Thank you for your suggestion. Additional proofreading was performed by professional native editor and the certificate for proofreading will be uploaded to the submission process.

**Reviewer #1**
**1) In the study population section, "Patients with images demonstrating evidence of distant metastatic disease were re-staged and pathologic confirmation for site of distant metastasis had tried in available cases." Please revise the wording "had tried" to be more clear.**

**Response:**

Thank you for your comment.

The sentence was revised to “Patients with images demonstrating evidence of distant metastatic disease were re-staged, and biopsy confirmation at the site of distant metastasis was attempted in anatomically accessible cases”

We have included this revision in the study population section.
